# Jasmonate-responsive MYB factors spatially repress rutin biosynthesis in *Fagopyrum tataricum*

**DOI:** 10.1093/jxb/ery032

**Published:** 2018-01-31

**Authors:** Kaixuan Zhang, Maria D Logacheva, Yu Meng, Jianping Hu, Dongpu Wan, Long Li, Dagmar Janovská, Zhiyong Wang, Milen I Georgiev, Zhuo Yu, Fuyu Yang, Mingli Yan, Meiliang Zhou

**Affiliations:** 1 Institute of Crop Science, Chinese Academy of Agricultural Sciences, Beijing, China; 2 Department of Evolutionary Biochemistry, AN Belozersky Institute of Physico-Chemical Biology, MV Lomonosov Moscow State University, Moscow, Russia; 3 College of Landscape and Travel, Agricultural University of Hebei, Baoding, China; 4 College of Agricultural Science, Xichang University, Xichang, Sichuan, China; 5 College of Agriculture, Inner Mongolia Agricultural University, Hohhot, Inner Mongolia, China; 6 School of Life Sciences, Hunan University of Science and Technology, Xiangtan, Hunan, China; 7 Department of Gene Bank, Crop Research Institute, Drnovská, Czech Republic; 8 College of Agriculture, Hainan University, Haikou, Hainan, China; 9 Group of Plant Cell Biotechnology and Metabolomics, The Stephan Angeloff Institute of Microbiology, Bulgarian Academy of Sciences, Plovdiv, Bulgaria; 10 Center of Plant Systems Biology and Biotechnology, Plovdiv, Bulgaria; 11 Grassland Institute, China Agricultural University, Beijing, China

**Keywords:** 26S proteasome, buckwheat, *Fagopyrum*, *tataricum*, jasmonates, MYB transcription factor, phenylpropanoid pathway

## Abstract

Jasmonates are plant hormones that induce the accumulation of many secondary metabolites, such as rutin in buckwheat, via regulation of jasmonate-responsive transcription factors. Here, we report on the identification of a clade of jasmonate-responsive subgroup 4 MYB transcription factors, FtMYB13, FtMYB14, FtMYB15, and FtMYB16, which directly repress rutin biosynthesis in *Fagopyrum tataricum*. Immunoblot analysis showed that FtMYB13, FtMYB14, and FtMYB15 could be degraded via the 26S proteasome in the COI1-dependent jasmonate signaling pathway, and that this degradation is due to the SID motif in their C-terminus. Yeast two-hybrid and bimolecular fluorescence complementation assays revealed that FtMYB13, FtMYB14, and FtMYB15 interact with the importin protein Sensitive to ABA and Drought 2 (FtSAD2) in stem and inflorescence. Furthermore, the key repressor of jasmonate signaling FtJAZ1 specifically interacts with FtMYB13. Point mutation analysis showed that the conserved Asp residue of the SID domain contributes to mediating protein–protein interaction. Protoplast transient activation assays demonstrated that FtMYB13, FtMYB14, and FtMYB15 directly repress phenylalanine ammonia lyase (*FtPAL*) gene expression, and FtSAD2 and FtJAZ1 significantly promote the repressing activity of FtMYBs. These findings may ultimately be promising for further engineering of plant secondary metabolism.

## Teaser

Jasmonate-responsive repressors of FtMYBs can be degraded by the 26S proteasome in a COI1-dependent manner in *Fagopyrum tataricum* and act together with FtSAD2 or FtJAZ1 in the phenylpropanoid pathway.

## Introduction

Buckwheat is a type of pseudocereal of the genus *Fagopyrum,* within the Polygonaceae family. Three major species, tartary buckwheat (TB; *Fagopyrum tataricum*), common buckwheat (CB; *Fagopyrum esculentum*), and golden buckwheat (*Fagopyrum cymosum*), are widely cultivated in Asia and Europe (Zhang *et al.*, 2012). Buckwheat can grow in harsh climates and nutrient-poor soils, which suggests that it has great ecological adaptability. Recently, TB research interest has increased due to its higher content of bioactive flavonoids (e.g. rutin, orientin, vitexin, quercetin, isovitexin, and isoorientin) compared with CB, and hence its potential to contribute to diverse health benefits. For instance, the concentration of rutin, a major flavonoid, can reach 81 mg g^–1^ in the groats of TB compared with the concentration of 0.2 mg g^–1^ reported in CB (Wijngaard and Arendt, 2006). Additionally, TB flour accumulates ~10-fold more total flavonoids than CB (Qin *et al.*, 2010). Therefore, buckwheat was considered to be a model plant for studying flavonoid biosynthesis due toits high accumulation of rutin, especially in the early days of research.

MYB transcription factors (TFs) are key regulators of plant flavonoid biosynthesis (Du *et al.*, 2010; Zhou and Memelink, 2016). MYB TFs are classified into four subgroups (R2R3-, 1R-, 3R-, and 4R-MYBs) on the basis of the number of adjacent repeats in their DNA-binding domains (Dubos *et al.*, 2010). Most of the MYB TFs that are involved in the regulation of flavonoid biosynthesis belong to the large R2R3-MYB family (Du *et al.*, 2010). In *Arabidopsis thaliana*, PAP1/MYB75, PAP2/MYB90, MYB112, MYB113, and MYB114 act positively in the flavonoid biosynthesis pathway (Zimmermann *et al.*, 2004; Teng *et al.*, 2005; Gonzalez *et al.*, 2008; Lotkowska *et al.*, 2015). However, MYB3, MYB4, MYB7, and MYB32, which are classified into subgroup 4 (S4) of the R2R3-MYB TFs, repress phenylpropanoid biosynthesis owing to the EAR repression motif (pdLHLD/LLxiG/S) and the conserved LLsrGIDPxT/SHRxI/L motif in their C-termini (Jin *et al.*, 2000; Preston *et al.*, 2004; Dubos *et al.*, 2010; Fornalé *et al.*, 2014; Zhou *et al.*, 2015*a*; Zhou *et al.*, 2017). It has been reported that MYB4, MYB7, and MYB32 interact with the importin β-like protein SAD2 (Sensitive to ABA and Drought 2) via a conserved GY/FDFLGL motif [the SAD2 Interaction Domain (SID) motif], mediating the transport of MYBs into the nucleus (Zhou *et al.*, 2015*a*). Recently, our group showed that the repression activity of MYB3 on expression of the *C4H* (cinnamate 4-hydroxylase) gene is directly regulated by the co-repressors NIGHT LIGHT-INDUCIBLE AND CLOCK-REGULATED 1 (LNK1) and LNK2 in phenylpropanoid biosynthesis (Zhou *et al.*, 2017). In *Brassica rapa* subsp. *rapa* cv. Tsuda turnip, BrMYB4, the ortholog of AtMYB4, acts as a negative regulator in the phenylpropanoid biosynthesis pathway because of its C-terminal repression motif (Zhang *et al.*, 2014). In buckwheat, FtMYB1 and FtMYB2 function positively in the accumulation of proanthocyanidins (Bai *et al.*, 2014).

Jasmonates (JAs) are important plant hormones that induce the biosynthesis of various secondary metabolites, such as flavonoids and anthocyanins, via modulation of JA-responsive TFs (Zhou and Memelink, 2016). The key transcriptional repressors, jasmonate ZIM domain (JAZ) proteins, can interact with several TFs with different roles in regulating JA-responsive gene expression (Yan *et al.*, 2007; Chini *et al.*, 2007; Thines *et al.*, 2007). A variety of MYB TFs have been found to interact with members of the JAZ family. These include the R2R3-MYB activators MYB21 and MYB24, which are involved in stamen development and male fertility (Song *et al.*, 2011), and MYB75, which is involved in anthocyanin biosynthesis and trichome initiation (Qi *et al.*, 2011). It has been also reported that JAs could boost phenylpropanoid biosynthesis in buckwheat (Kim *et al.*, 2011). However, the JA-regulated mechanism of the phenylpropanoid biosynthetic pathway is still unknown in buckwheat.

Here, a clade of JA-responsive MYB repressors, including FtMYB13, FtMYB14, FtMYB15, and FtMYB16, are described in relation to their role in phenylpropanoid biosynthesis. We found that these MYB repressors spatially regulate phenylpropanoid biosynthesis at the transcript and the protein level in different tissues. In addition, the repressing activity of FtMYBs is directly enhanced by FtSAD2 or the co-repressor FtJAZ1 via protein–protein interaction.

## Materials and methods

### Isolation of *FtMYB13*, *FtMYB14*, *FtMYB15,* and *FtMYB16* genes and the *FtPAL* promoter

The publicly available database of *F. tataricum* genes in the flower and inflorescence and a genome database were collected (Logacheva *et al.*, 2011). Searches for DNA sequences containing a conserved MYB domain were performed to confirm the candidate MYB genes. In addition, SMARTRACE and PCR technology were utilized to clone the full-length FtMYB13, FtMYB14, FtMYB15, and FtMYB16 transcripts with the following primers: 5ʹ-ATGGGAAGAGCTCCTTGTTGC-3ʹ and 5ʹ-TCAGATGAGCAAAGACTCAGC-3ʹ for *FtMYB13*; 5ʹ-ATGG GTAGATCTCCATGTTGTG-3ʹ and 5ʹ-TCATTTCATCTCCAATG ATC-3ʹ for *FtMYB14*; 5ʹ -ATGGGTCGATCTCCATGTTGC-3ʹ and 5ʹ-TCATTTCATCTCCAAAGTTCTATAG-3ʹ for *FtMYB15*; 5ʹ-ATG GGGAGATCACCTTGCTGC-3ʹ and 5ʹ-CTAGCTTGTTGTGGCAT TAGAAG-3ʹ for *FtMYB16*; 5ʹ-ATGGATCTTCCAAGCCTCGCT-3ʹ and 5ʹ-TCAAGAGAGTTCTTCGAGCAT-3ʹ for *FtSAD2*; 5ʹ-ATGA ACTTGTTCCCACTGAAAG-3ʹ and 5ʹ-TCAGGGCTGAATCGACG TCCG-3ʹ for *FtJAZ1.* The *FtPAL* promoter fragments corresponding to the sequence from the transcription start site to 974 bp upstream of the transcription start site were isolated using the primers FtPALproF: 5ʹ-GTCGTTAAATATCGTTAAAAT-3ʹ and FtPALproR: 5ʹ-CCACCCCAACGGATCCTGCAC-3ʹ. The RNA isolation, cDNA synthesis, amplification conditions and sequence analysis were as described previously (Zhou *et al.*, 2015*b*).

### Plant materials, growth conditions, and chemical treatments

Two-week-old *F. tataricum* seedlings and 20-day-old hairy root lines were treated with 50 µM MeJA (Sigma-Aldrich, St. Louis, MO, USA) as described previously (Zhou *et al.*, 2015*b*). For the construction of transgenic *F. tataricum* hairy root lines constitutively overexpressing *FtMYB13-HA*, *FtMYB13*^*D281N*^*-HA*, *FtMYB13*^*D283N*^*-HA*, *FtMYB13*^*D285N*^*-HA*, *FtMYB14-HA*, *FtMYB14*^*D266N*^*-HA*, *FtMYB15-HA*, *FtMYB15*^*D258N*^*-HA*, *FtMYB16-HA*, *FtSAD2-HA*, and *FtJAZ1-HA*, pRT101 plasmids containing these genes were digested with *Sph*I and the genes were subsequently cloned into pCAMBIA1300. The binary vectors pCAMBIA1300-*FtMYB13-HA*, pCAMBIA1300-*FtMYB13*^*D281N*^*-HA*, pCAMBIA1300-*FtMYB13*^*D283N*^*-HA*, pCAMBIA 1300-*FtMYB13*^*D285N*^*-HA*, pCAMBIA1300-*FtMYB14-HA*, pCAMBIA1300- *FtMYB14*^*D266N*^*-HA*, pCAMBIA1300-*FtMYB15-HA*, pCAMBIA1300-*FtMYB15*^*D258N*^*- HA*, pCAMBIA1300-*FtMYB16-HA*, pCAMBIA1300-*FtSAD2-HA*, and pCAMBIA1300-*FtJAZ1-HA* were introduced into the *Agrobacterium rhizogenes* A4 strain (agropine type). Sterile *F. tataricum* leaves were cut into squares and transfected with *A. rhizogenes* A4 strains containing the above plasmids. Roots developed at the cut edges 2–3 weeks after co-cultivation. *F. tataricum* transgenic hairy roots were identified using the methods described by Zhou *et al.* (2010). All primer sequences are listed in Supplementary Table S1 at *JXB* online.

### Yeast two-hybrid assays

The full-length genes *FtMYB13*, *FtMYB13*^*D281N*^, *FtMYB13*^*D283N*^, *FtMYB13*^*D285N*^, *FtMYB14*, *FtMYB14*^*D266N*^, *FtMYB15*, *FtMYB15*^*D258N*^, *FtMYB16*, *FtSAD2*, and *FtJAZ1* were cloned into pACT2 or pAS2.1 (James *et al.*, 1996). Point mutations of the genes were generated using the GeneTailor^TM^ Site-Directed Mutagenesis System (Life Technologies; http://www.thermofisher.com). The PCR fragments and constructed plasmids were confirmed by sequencing. Co-transformation with bait and prey plasmids at a ratio of 1:1 was performed in yeast strain PJ69-4A according to a modified yeast transformation protocol (Gietz *et al.*, 1992). All primer sequences are listed in Supplementary Table S1.

### Yeast one-hybrid assays

*FtMYB13*, *FtMYB14*, *FtMYB15*, and *FtMYB16* were PCR amplified from pACT2-FtMYB13, pACT2-FtMYB14, pACT2-FtMYB15, and pACT2-FtMYB16, digested with *Bam*HI and *Pst*I, and cloned into pAS2.1. The plasmids were introduced into the yeast strain PJ69-4A with the reporter genes *His3* and *LacZ* following the manufacturer’s instructions (Clontech, Palo Alto, CA, USA). The promoter fragment of *FtPALpro* was digested with *Not*I and *Sma*I and fused to a TATA box-*HIS3* gene in the plasmid pHIS3NX. *HIS3* gene constructs were integrated into the genome of yeast strain Y187. After transformation with the plasmid pACT2-FtMYB13, pACT2-FtMYB14, pACT2-FtMYB15, or pACT2-FtMYB16, yeast cells were grown on minimal synthetic defined (SD)-glucose medium lacking Leu and His (SD/-LH) supplemented with 3-amino-1,2,4-triazole (3-AT; Sigma) at increasing concentrations ranging from 0 to 50 mM. Yeast cells containing the empty pAS2.1 vector were used as a negative control. The colony-lift filter β-galactosidase assay was carried out according to the Yeast Protocols Handbook (Clontech Laboratories, Inc.). All primer sequences are listed in Supplementary Table S1.

### Confocal microscopy

To observe the subcellular localization of *FtMYB13*, *FtMYB13*^*D281N*^, *FtMYB13*^*D283N*^, *FtMYB13*^*D285N*^, *FtMYB14*, *FtMYB14*^*D266N*^, *FtMYB15*, *FtMYB15*^*D258N*^, and *FtMYB16*, the green fluorescent protein (GFP)-fused open reading frames were cloned into pTH2. For bimolecular fluorescence complementation (BiFC) assays, *FtMYB13*, *FtMYB13*^*D281N*^, *FtMYB13*^*D283N*^, *FtMYB13*^*D285N*^, *FtMYB14*, *FtMYB14*^*D266N*^, *FtMYB15*, *FtMYB15*^*D258N*^ and *FtMYB16*, *FtSAD2*, and *FtJAZ1* were cloned into pRTL2-YNEE (nYFP-) or pRTL2-HAYC (-cYFP). All constructs were transiently expressed or co-expressed with all possible combinations of nYFP and cYFP fusion proteins by polyethylene glycol (PEG)-mediated transfection into Arabidopsis protoplasts, as previously described (Schirawski *et al.*, 2000). Microscopic images of transfected protoplasts were acquired with a Leica DM IRBE confocal laser scanning microscope and analyzed with ImageJ (Abràmoff *et al.*, 2004). Primers used in the BiFC assays are listed in Supplementary Table S1.

### Arabidopsis protoplast transactivation assays

The promoter fragment of *FtPAL* was amplified on genomic DNA and cloned into the reporter plasmid pGusXX (Pasquali *et al.*, 1994). The *FtMYB13*, *FtMYB13*^*D281N*^, *FtMYB13*^*D283N*^, *FtMYB13*^*D285N*^, *FtMYB14*, *FtMYB14*^*D266N*^, *FtMYB15*, *FtMYB15*^*D258N*^ and *FtMYB16*, *FtSAD2*, and *FtJAZ1* were PCR amplified and cloned into the effector plasmid pRT101 (Töpfer *et al.*, 1987). Protoplasts isolated from an Arabidopsis cell suspension were co-transformed with a reporter plasmid carrying the *FtPAL* promoter fused to the β-glucuronidase gene (*GUS*) and effector plasmids carrying either the *FtMYB13*, *FtMYB13*^*D281N*^, *FtMYB13*^*D283N*^, *FtMYB13*^*D285N*^, *FtMYB14*, *FtMYB14*^*D266N*^, *FtMYB15*, *FtMYB15*^*D258N*^, *FtMYB16*, *FtSAD2*, or *FtJAZ1* gene fused to the CaMV 35S promoter. As controls, co-transformations of *FtPAL*-promoter-GUS with the empty vector pRT101 were used. PEG-mediated transfection of protoplasts and GUS activity assays were performed as described previously (van der Fits and Memelink, 1997; Schirawski *et al.*, 2000). All primers used in the protoplast transactivation assays are listed in Supplementary Table S1.

### Quantitative RT–PCR

Reverse transcription of total RNA was carried out by using the Revert AidTM First Strand cDNA Synthesis Kit (Fermentas). The primers of *FtPAL*, *FtC4H*, *Ft4CL*, *FtCHS*, *FtCHI*, *FtF3H*, *FtF3ʹH*, *FtFLS*, and the reference gene *FtH3* for quantitative real-time (qRT)–PCR were as previously described (Li *et al.*, 2010; Zhou *et al.*, 2015*b*). The qRT–PCR amplification was performed as described in previous reports (Zhou *et al.*, 2010; Zhou *et al.*, 2015*b*), and the result was visualized as a heat map generated by TreeView 1.1.3.

### Protein extraction and western blot

Protein extraction from *F. tataricum* hairy roots and immunoblot analysis with anti-hemagglutinin (HA) or anti-GFP peroxidase antibodies followed the methods described by Zhou *et al.* (2015a, b).

### Rutin measurement

Different tissues of 60-day-old *F. tataricum* plants were harvested and freeze-dried. *F. tataricum* hairy roots (1 g) were harvested, frozen, and ground in liquid nitrogen. Frozen samples were extracted twice in 50 ml methanol for 24 h at 4 °C and extracts were vacuum-dried at 80 °C and dissolved in 10 ml methanol. The solution was then filtered through a polyvinylidene filter (pore size 0.45 μm) and diluted two-fold with methanol. The concentration of rutin was analyzed by high-performance liquid chromatography (HPLC) of triplicate independent extractions for each line, as described elsewhere (Huang *et al.*, 2016).

### Statistical analysis

All data were analyzed using Student’s *t*-test and one-way ANOVA. Values of *P*<0.05 were considered to be signiﬁcant.

## Results

### Characterization of JA-responsive R2R3-MYB S4 family members

**Fig. 1.**
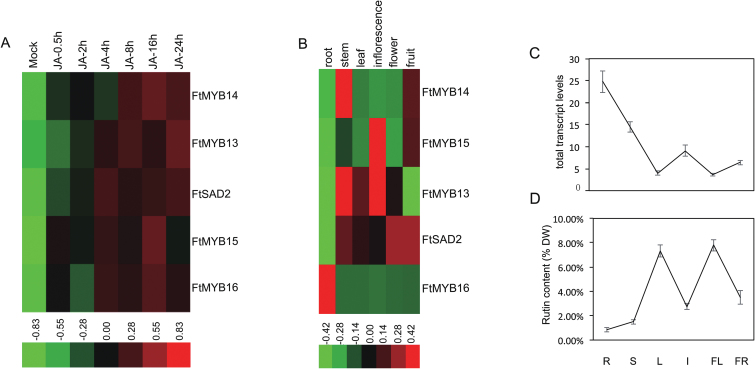
Characterization of JA-responsive members of the R2R3-MYB S4 TF family. (A) Expression pattern of *FtMYB13*, *FtMYB14*, *FtMYB15*, and *FtMYB16* in 2-week-old *F. tataricum* seedlings in response to exposure to 50 µM MeJA for various times (in h) as indicated. *FtH3* was used as an internal control. Mock indicates exposure to JA for 0 h. (B) Expression patterns of *FtMYB13*, *FtMYB14*, *FtMYB15*, and *FtMYB16* in different tissues, as detected by qRT–PCR. Heat maps were generated by hierarchical clustering based on Pearson’s correlation. Different shades indicate higher or lower expression. (C) Measurement of total transcript levels of *FtMYB13*, *FtMYB14*, *FtMYB15*, and *FtMYB16* in different tissues by qRT–PCR. (D) Measurement of rutin concentration in different tissues by HPLC. Values are the mean±SD of three biological repeats. DW, Dry weight; FL, flower; FR, fruit; I, inflorescence; L, leaf; R, root; S, stem.

To identify the members of the buckwheat R2R3-MYB S4 family, a BLAST search of the CB genome database (Yasui *et al.*, 2016) and TB transcripts database (Logacheva *et al.*, 2011) was performed, using the EAR domain of MYB4 from Arabidopsis as a query sequence (Zhou *et al.*, 2015*a*). Of these sequences, *FtMYB13* (GenBank accession no. KY290579), *FtMYB14* (GenBank accession no. KY290580), *FtMYB15* (GenBank accession no. KY290581), and *FtMYB16* (GenBank accession no. KY290582) contained an EAR motif (Supplementary Fig. S1) and shared the highest identity with subgroup 4 TFs (Supplementary Fig. S2; Jin *et al.*, 2000; Zhou *et al.*, 2015*a*). To gain a general perspective on the amount of *FtMYBs* transcripts response to JAs, qRT–PCR was performed. As shown in Fig. 1A, *FtMYB13* mRNA accumulation was highly induced by MeJA treatment and reached its highest level at 24 h, while *FtMYB14*, *FtMYB15* and *FtMYB16* were slightly induced. The spatial transcript accumulation analysis showed that the highest level of *FtMYB13* was in the stem, leaves, and inflorescence, while the highest level of *FtMYB14* was in stem, and that of *FtMYB15* was in inflorescence (Fig. 1B). It is noteworthy that the highest level of *FtMYB16* was observed in root; levels of this gene were relatively low in the other tissues studied, and none of the other genes showed high levels in root (Fig. 1B). In 60-day-old *F. tataricum* plants, the mRNA accumulation of four *FtMYBs* reached its highest level in root and the lowest level in leaf and flower (Fig. 1C), while the largest amount of rutin was found in leaf and flower, and the lowest amount in root (Fig. 1D). The mRNA accumulation of the activators *FtMYB1* and *FtMYB2* showed similar levels in different tissues of TB (Bai *et al.*, 2014). These results indicate that rutin biosynthesis is probably spatially regulated by a clade of R2R3-MYB repressors, FtMYB13, FtMYB14, FtMYB15, and FtMYB16.

### FtMYB13, FtMYB14, and FtMYB15 are JA-responsive nuclear-localized repressors

**Fig. 2.**
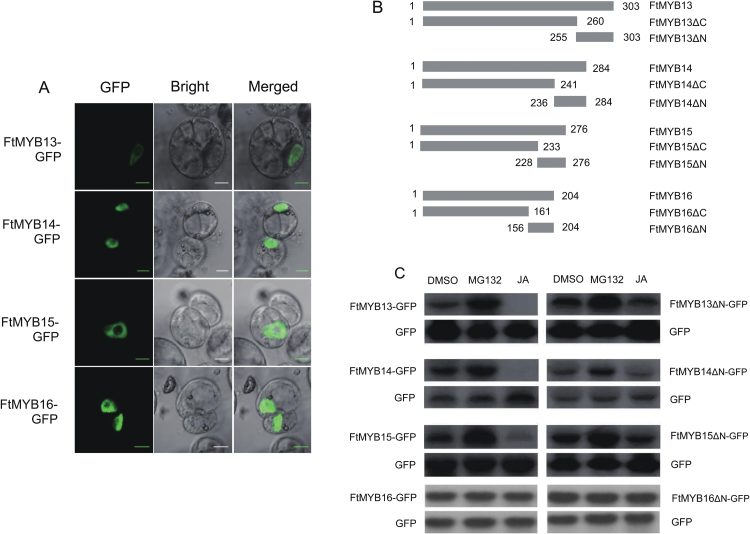
FtMYB13, FtMYB14, and FtMYB15 are JA-responsive, nuclear-localized repressors. (A) A FtMYB13-GFP, FtMYB14-GFP, FtMYB15-GFP, or FtMYB16-GFP construct was transformed into protoplasts isolated from an Arabidopsis cell suspension and examined by confocal laser scanning microscopy. The confocal microscopic image is shown at the left (GFP), the corresponding differential interference contrast (Bright) image is in the middle, and the merged image is at the right. Scale bar=20 µm. (B) Schematic diagram of the N- and C-terminus of FtMYB13, FtMYB14, FtMYB15, and FtMYB16 proteins. (C) Immunoblot analysis with anti-GFP antibodies of total protein extracts from Arabidopsis cell suspension protoplasts transiently co-expressing GFP and FtMYB13-GFP, FtMYB14-GFP, FtMYB15-GFP, FtMYB16-GFP, FtMYB13ΔN-GFP, FtMYB14ΔN-GFP, FtMYB15ΔN-GFP, or FtMYB16ΔN-GFP. Arabidopsis protoplasts were harvested 18 h after transformation of protoplasts treated for 4 h with DMSO at 0.1% (v/v) final concentration, with 50 µM of the 26S proteasome inhibitor MG132, or with 50 µM MeJA.

The above data suggest that FtMYB13, FtMYB14, FtMYB15, and FtMYB16 may be nuclear-localized repressors of the rutin biosynthetic pathway (Fig. 1). To examine this hypothesis, the gene encoding GFP was fused to *FtMYB13*, *FtMYB14*, *FtMYB15*, or *FtMYB16*, positioned so that GFP would be expressed at the C-terminus of the respective protein, with each construct under the control of the CaMV 35S promoter. The resulting plasmid constructs were introduced into Arabidopsis protoplasts. As shown in Fig. 2A, all of these MYB factors were localized in the nucleus, as hypothesized. Transcriptional activity assays in the yeast PJ69-4A strain suggested that only transformants containing pAS2.1 (GAL4-BD: GAL4 DNA binding domain) that were fused with FtMYBs grew well on SD-glucose medium lacking Trp (SD/-W) plates. The β-galactosidase activity assay consistently showed that the β-galactosidase activity of the transformant with pAS2.1-FtMYBs was ~2-fold lower than that of pAS2.1 (Supplementary Fig. S3), demonstrating that these FtMYBs repressed the expression of the reporter gene *LacZ* in the genome of PJ69-4A.

As shown in Fig. 1, transcripts of *FtMYB*s were induced by JAs. This finding could not exclude the possibility that JAs also affect the activity of FtMYBs at the protein level. Arabidopsis protoplasts were co-transformed with a GFP-expressing plasmid and a plasmid expressing a FtMYB-GFP fusion, and the transfected protoplasts were treated for 4 h with MeJA or the 26S proteasome inhibitor MG132 or the solvent DMSO. Immunoblot analysis of total cellular protein with anti-GFP antibodies revealed that MG132 increased and MeJA decreased the accumulation of FtMYB13-GFP, FtMYB14-GFP, and FtMYB15-GFP drastically (Fig. 2C), indicating that these TFs are subject to JA-related 26S proteasome-mediated degradation. However, neither MeJA nor MG132 treatment influenced the abundance of GFP and FtMYB16-GFP. Furthermore, we examined the role of COI1, the receptor of the JA signaling pathway (Yan *et al.*, 2009), in JA-induced degradation of FtMYBs. In *coi1-1* leaf protoplasts, MG132 and MeJA did not affect the amount of FtMYB13-GFP, FtMYB14-GFP, or FtMYB15-GFP fusion protein, indicating that JA-induced degradation of FtMYBs is COI1 dependent (Supplementary Fig. S4). Our previous study showed that the C-terminal of the R2R3-MYB S4 TFs performs the regulatory activity (Zhou *et al.*, 2015*a*). To determine whether the C-terminus of FtMYBs is responsible for JA-induced degradation, we constructed plasmids expressing the C-terminus and the N-terminus of FtMYBs fused to GFP (Fig. 2B). The results showed that MG132 and MeJA still affected the levels of FtMYB13ΔN-GFP, FtMYB14ΔN-GFP, and FtMYB15ΔN-GFP protein (Fig. 2C); however, FtMYB13ΔC-GFP, FtMYB14ΔC-GFP, and FtMYB15ΔC-GFP were not subject to MeJA-mediated degradation (Supplementary Fig. S10), demonstrating that the C-terminus of FtMYB13, FtMYB14, and FtMYB15 contain the degron responsive to JAs. Taken together, these results indicate that FtMYB13, FtMYB14, and FtMYB15 are JA-responsive, nuclear-localized transcriptional repressors.

### Asp to Asn mutation of the SID domain releases FtMYBs from interaction with FtSAD2

**Fig. 3.**
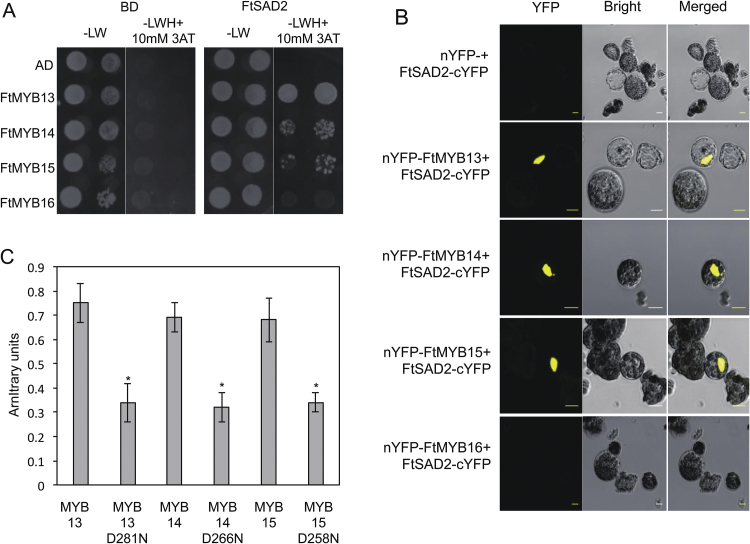
The Asp to Asn mutation of the SID domain releases FtMYBs from FtSAD2 interaction. (A) FtMYB13, FtMYB14, and FtMYB15 interact with FtSAD2 in yeast. Yeast cells expressing FtMYB13, FtMYB14, FtMYB15, and FtMYB16 proteins fused to the GAL4 AD, and FtSAD2 fused to the GAL4 BD, were spotted on SD/-LW medium to select for the plasmids, and on SD/-LWH medium with 10 mM 3-AT to select for transcriptional activation of the *His3* gene. Growth was monitored after 7 days. Yeast cells transformed with the empty plasmids pAS2.1 and pACT2, expressing GAL4 BD and GAL4 AD, respectively, were used as controls. (B) BiFC assays *in planta*. YFP fluorescence images (alone or merged with bright-field images) of Arabidopsis cell suspension protoplasts co-transformed with constructs encoding the indicated fusion proteins with YFP at the N-terminus (nYFP) or the C-terminus (cYFP). Scale bar=20 µm. (C) Interaction of FtMYB13^D281N^, FtMYB14^D266N^, and FtMYB15^D258N^ with FtSAD2 in quantitative Y2H assays. A liquid culture β-galactosidase assay was performed on the transformed yeasts. The activity of β-galactosidase was measured in arbitrary units. Values are the mean±SD of three biological repeats. Asterisks indicate statistically signiﬁcant differences compared with FtMYB13, FtMYB14, and FtMYB15 (*P*<0.05, Student’s *t*-test).

Our previous results showed that R2R3-MYB S4 TFs interact with SAD2 protein via the SID domain in their C-terminus (Zhou *et al.*, 2015*a*). FtMYB13, FtMYB14, and FtMYB15 possess a conserved SID or SID-like domain (Supplementary Figs S1 and S5); thus, we hypothesized that these three FtMYBs may interact with FtSAD2 as well. Yeast two-hybrid (Y2H) assays were used as a first step to test the interaction between FtMYB13, FtMYB14, FtMYB15, or FtMYB16 and FtSAD2. Results showed that yeast cells co-expressing FtSAD2 and FtMYB13, FtMYB14, or FtMYB15 were able to sustain growth at 3-AT concentrations up to 10 mM on selective medium (Fig. 3A). However, no interaction was detected between FtSAD2 and FtMYB16. To confirm the results of the Y2H assays, BiFC assays were performed. Strong fluorescent signals were observed in the nucleus of Arabidopsis protoplasts upon co-expression of nYFP-FtMYB13 and FtSAD2-cYFP, nYFP-FtMYB14 and FtSAD2-cYFP, or nYFP-FtMYB15 and FtSAD2-cYFP, respectively (Fig. 3B). No or only background YFP fluorescence was detected in negative controls (nYFP co-expressed with FtSAD2-cYFP) and with co-expression of FtSAD2-cYFP and nYFP-FtMYB16. These results indicate that FtSAD2 interacts with FtMYB13, FtMYB14, and FtMYB15 in the nucleus.

It has been demonstrated that the conserved Asp residue of the SID motif contributes to the protein–protein interaction (Zhou *et al.*, 2015*a*). Thus, we hypothesized that the conserved Asp residue (FtMYB13Asp281, FtMYB14Asp266, FtMYB15Asp258) could be essential for the interaction of FtMYB13, FtMYB14, or FtMYB15 with FtSAD2. As the first step to test this hypothesis, we generated derivatives of these FtMYBs, including FtMYB13^D281N^, FtMYB14^D266N^, and FtMYB15^D258N^, by introducing point mutations, and we then used these mutants in Y2H assays. As shown in Fig. 3C, in quantitative Y2H assays the interaction of FtMYB13^D281N^, FtMYB14^D266N^, and FtMYB15^D258N^ with FtSAD2 was significantly diminished, indicating that these FtMYB derivatives had lost the ability to interact with FtSAD2 protein.

The importin β-like protein SAD2 can mediate the nuclear localization of MYBs in Arabidopsis (Zhou *et al.*, 2015*a*). Therefore, FtMYB13^D281N^, FtMYB14^D266N^, and FtMYB15^D258N^ protein may be absent from the nucleus. To test this hypothesis, subcellular localization analysis was performed. As expected, FtMYB13^D281N^-GFP, FtMYB14^D266N^-GFP, and FtMYB15^D258N^-GFP protein were found only in the cytoplasm (Supplementary Fig. S6). These results suggest that the conserved Asp residue of the SID domains of FtMYB13, FtMYB14, and FtMYB15 are necessary for their transport into the nucleus mediated by FtSAD2. As shown in Fig. 1, FtSAD2 transcription was also induced by JA and transcripts accumulated in stem, leaves and flowers, similar to the pattern of transcript accumulation of *FtMYB13*, *FtMYB14*, and *FtMYB15*. This indicates that FtSAD2 probably directly regulates the transcriptional activity of these FtMYBs.

### FtMYB13 interacts with the JA-responsive repressor FtJAZ1

**Fig. 4.**
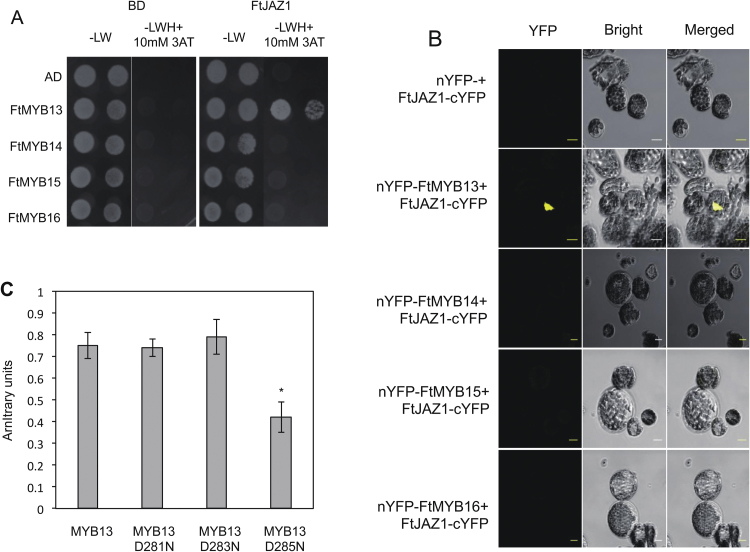
FtMYB13 interaction with FtJAZ1 depends on a conserved Asp residue. (A) FtMYB13 interacts with FtJAZ1 in yeast. Yeast cells expressing FtMYB13, FtMYB14, FtMYB15, or FtMYB16 proteins fused to the GAL4 AD, and FtJAZ1 fused to the GAL4 BD, were spotted on SD/-LW medium to select for the plasmids, and on SD/-LWH medium with 10 mM 3-AT to select for transcriptional activation of the *His3* gene. Growth was monitored after 7 days. Yeast cells transformed with the empty plasmids pAS2.1 and pACT2, expressing GAL4 BD and GAL4 AD, respectively, were used as controls. (B) FtMYB13 interacts with FtJAZ1 *in planta*. YFP fluorescence images (alone or merged with bright-field images) of Arabidopsis cell suspension protoplasts co-transformed with constructs encoding the indicated fusion proteins with YFP at the N-terminus (nYFP) or the C-terminus (cYFP). Scale bar=20 µm. (C) Interaction of FtMYB13^D281N^, FtMYB13^D283N^, and FtMYB13^D285N^ with FtJAZ1 in quantitative Y2H assays. A liquid culture β-galactosidase assay was performed on the transformed yeasts. The activity of β-galactosidase was measured in arbitrary units. Values are the mean±SD of three biological repeats. Asterisks indicate statistically signiﬁcant differences compared with FtMYB13, FtMYB13^D281N^, and FtMYB13^D283N^ (*P*<0.05, Student’s *t*-test).

In Arabidopsis, AtJAZ proteins interact with MYBs, modulating the expression of key enzyme genes, such as *C4H*, in the anthocyanin biosynthesis pathway (Qi *et al.*, 2011). As FtMYB13, FtMYB14, and FtMYB15 are sensitive to JAs at the transcript and protein levels, we assumed that these FtMYBs interact with FtJAZ protein in a similar manner. Interestingly, Y2H assays suggested that FtMYB13 was able to bind FtJAZ1, but FtMYB14, FtMYB15, and FtMYB16 did not show any interaction with FtJAZ1 (Fig. 4A). The same results were observed via BiFC assays (Fig. 4B). Furthermore, none of the FtMYBs interacted with FtJAZ2 (data not shown). The conserved SID domain of FtMYB13 possesses three conserved Asp residues (GL*D*F*D*L*D*L; Asp281, Asp283, and Asp285), among which Asp281 is essential for interaction with SAD2 (Fig. 3). Thus, we assume that Asp283 and Asp285 may be considered more specific for interaction with FtJAZ1. The interaction of FtMYB13^D285N^ with FtJAZ1, tested by quantitative Y2H assays, was significantly diminished (Fig. 4C), while FtMYB13^D281N^ and FtMYB13^D283N^ showed no difference relative to FtMYB13. Moreover, FtMYB13^D283N^-GFP and FtMYB13^D285N^-GFP proteins were found in the nucleus (Supplementary Fig. S6). In summary, the Asp285 to Asn285 mutation abolished the interaction between FtMYB13 and FtJAZ1.

### The transcriptional repression activity of FtMYBs is regulated by FtSAD2 or FtJAZ1

**Fig. 5.**
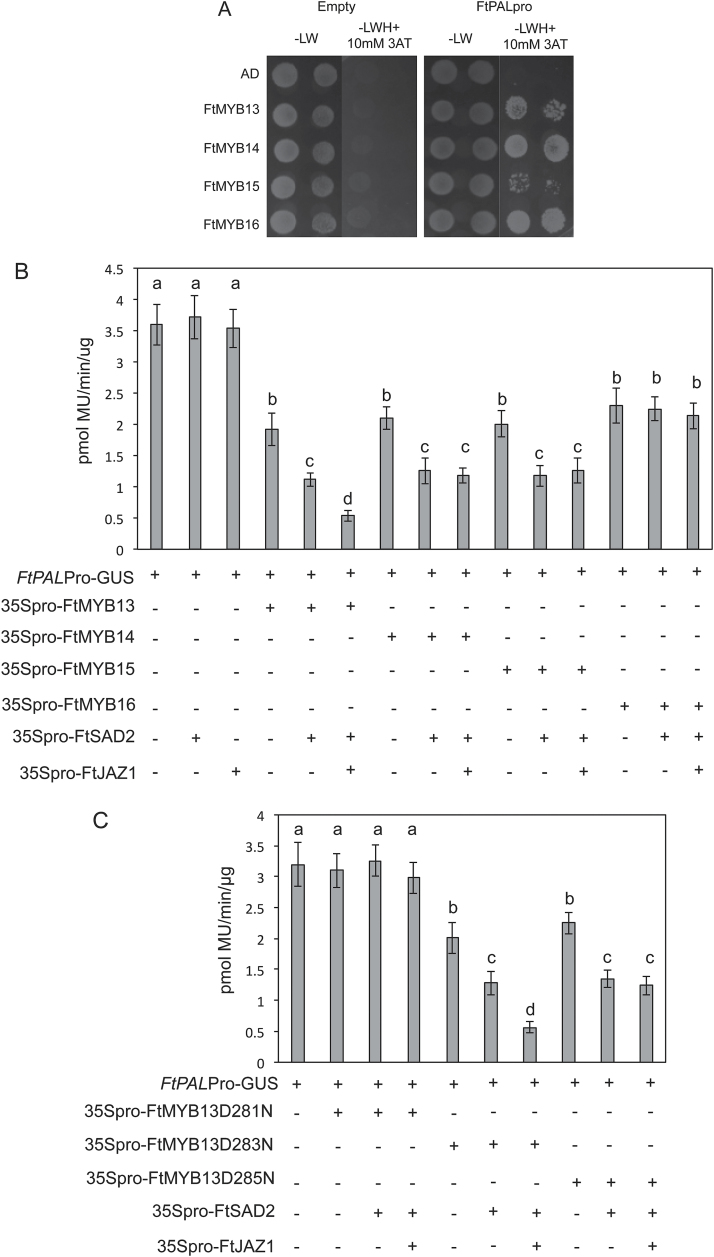
The repression activity of FtMYB13, FtMYB14, and FtMYB15 is modulated by FtSAD2 or FtJAZ1. (A) Y1H analysis of FtMYB13, FtMYB14, FtMYB15, and FtMYB16 binding to the *FtPALpro* fragment. Transformed yeasts (SD/-L) were grown on SD/-LH medium supplemented with 10 mM 3-AT. Yeast cells were allowed to grow for 7 days at 30 °C. (B, C) Protoplast transactivation assays. Arabidopsis protoplasts were co-transformed with 2 µg of a reporter construct of *FtPALpro*-GUS and 2 µg of effector plasmids. The effector constructs consisted of an expression vector carrying the CaMV 35S promoter with or without the *FtMYB13*, *FtMYB13*^*D281N*^, *FtMYB13*^*D283N*^, *FtMYB13*^*D285N*^, *FtMYB14*, *FtMYB15*, *FtMYB16*, *FtSAD2*, or *FtJAZ1* gene. Values represent the mean±SE of triplicate experiments. Significant differences between values are indicated with different letters (*P*<0.05, one-way ANOVA).

The first common step of the phenylpropanoid pathway is catalyzed by phenylalanine ammonia lyase (PAL). Analysis of the *FtPAL* gene promoter revealed a MYB binding element (AATAGTT). Yeast one-hybrid (Y1H) assays were performed to test the direct interaction between the FtMYBs and the *FtPAL* promoter. A 974 bp fragment of the *FtPAL* promoter was cloned into the pHIS3NX reporter vector, and its interaction with pACT2-FtMYB13, pACT2-FtMYB14, pACT2-FtMYB15, and pACT2-FtMYB16, harboring FtMYB13, FtMYB14, FtMYB15, and FtMYB16 [GAL4 activation domain (AD) fused], respectively, was assessed. As shown in Fig. 5A, these four MYBs could bind to the promoter of *FtPAL*.

To elucidate the functional significance of the interaction between the four FtMYBs and FtSAD2 or FtJAZ1, we co-transformed an Arabidopsis cell suspension with identical amounts of effector plasmids carrying FtMYB13, FtMYB13^D281N^, FtMYB13^D283N^, FtMYB13^D285N^, FtMYB14, FtMYB14^D266N^, FtMYB15, FtMYB15^D258N^, or FtMYB16 with FtSAD2 or FtJAZ1, or in combination with the *FtPALpro*-GUS reporter constructs. As shown in Fig. 5B and C, the repressing activity of FtMYB13, FtMYB13^D283N^, and FtMYB13^D285N^ on *FtPALpro*-GUS was significantly enhanced by FtSAD2 and FtJAZ1. In addition, FtSAD2 also significantly increased the repression activity of both FtMYB14 and FtMYB15. However, neither FtSAD2 nor FtJAZ1 affected the transcriptional activity of FtMYB13^D281N^, FtMYB14^D266N^, FtMYB15^D258N^, or FtMYB16 (Fig. 5B, C; Supplementary Fig. S7), indicating that they act dependently via protein–protein interaction. These results demonstrated that FtMYBs directly repress *FtPAL* expression, which is modulated by their interacting partners FtSAD2 and FtJAZ1.

### Overexpression of FtMYB13, FtMYB14, FtMYB15, or FtMYB16 represses rutin biosynthesis in *F. tataricum* hairy roots

**Fig. 6.**
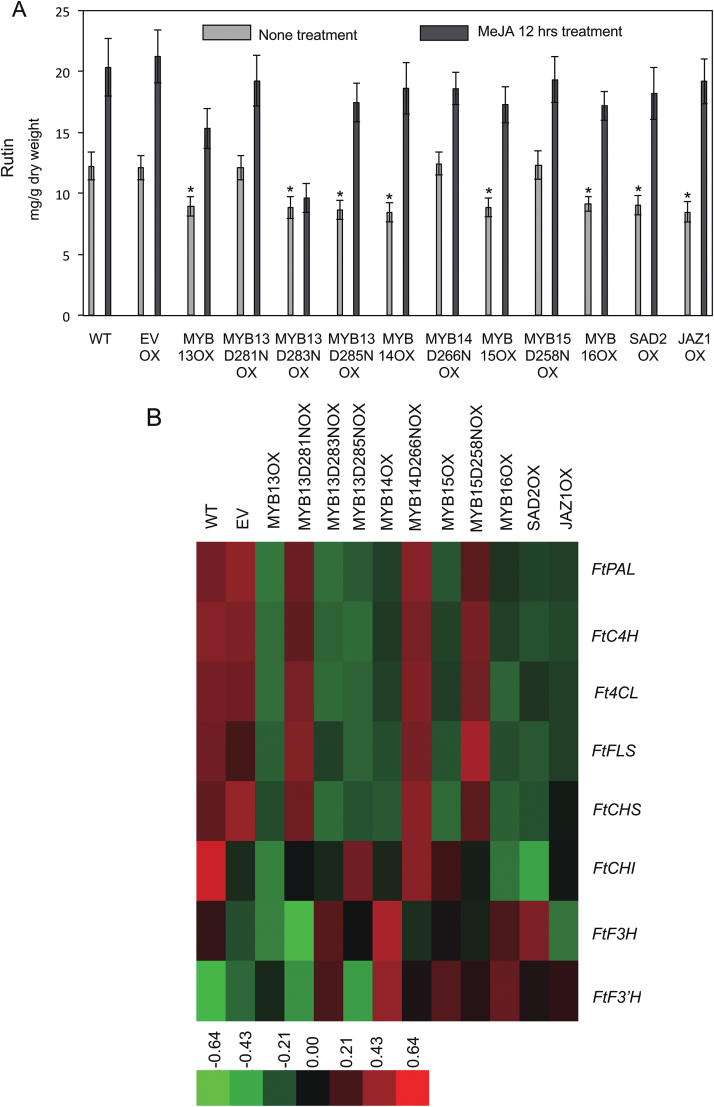
FtMYB13, FtMYB14, FtMYB15, and FtMYB16 represses rutin biosynthesis in *F. tataricum* hairy roots. (A) Rutin content in different genotypes of *F. tataricum* hairy roots with and without treatment for 12 h with 50 µM JA. WT, wild-type hairy roots. The transgenic hairy root lines were generated by transformation with strains of *A. rhizogenes* A4 harboring T-DNA containing no open reading frame (empty vector; EV), *FtMYB13*, *FtMYB13*^*D281N*^, *FtMYB13*^*D283N*^, *FtMYB13*^*D285N*^, *FtMYB14*, *FtMYB14*^*D266N*^, *FtMYB15*, *FtMYB15*^*D258N*^, *FtMYB16-HA*, *FtSAD2-HA*, or *FtJAZ1-HA*. Values are the mean±SE of three biological repeats of each independent transgenic line. Asterisks indicate statistically significant differences compared with WT and EV (*P*<0.05). (B) Expression levels of *FtPAL*, *FtC4H*, *Ft4CL*, *FtFLS*, *FtCHS*, *FtCHI*, *FtF3H*, and *FtF3ʹH* in different transgenic hairy root lines, as indicated. *FtH3* was used as an internal control.

The above data show that FtMYB13, FtMYB14, FtMYB15, and FtMYB16 repress the expression of *FtPAL*, suggesting that these factors probably regulate rutin biosynthesis. To functionally test the roles of these MYB TFs and their derivatives *in planta*, we investigated the accumulation of rutin in *F. tataricum* hairy roots overexpressing *FtMYB13-HA*, *FtMYB13*^*D281N*^*-HA*, *FtMYB13*^*D283N*^*-HA*, *FtMYB13*^*D285N*^*-HA*, *FtMYB14-HA*, *FtMYB14*^*D266N*^*-HA*, *FtMYB15-HA*, *FtMYB15*^*D258N*^*-HA*, *FtMYB16-HA*, *FtSAD2-HA*, or *FtJAZ1-HA.* Three independent transgenic hairy root lines of each genotype were identified by western blot (Supplementary Fig. S8) and used for further functional analysis. The hairy root growth occurred primarily during the first 5 days of cultivation, and the maximum content of rutin peaked at 12.15 mg g^–1^ dry weight at day 20 of cultivation. The 20-day-old hairy root lines were chosen to study the function of these FtMYBs in rutin accumulation. As shown in Fig. 6A, the rutin levels in the *FtMYB13*, *FtMYB13*^*D283N*^, *FtMYB13*^*D285N*^, *FtMYB14*, *FtMYB15*, and *FtMYB16* overexpressing lines were significantly lower than those of the wild-type (WT), empty vector (EV), and *FtMYB13*^*D281N*^, *FtMYB14*^*D266N*^, and *FtMYB15*^*D258N*^ overexpressing lines, ranging from 8.45 to 9.16 mg g^–1^ dry weight. MeJA treatment drastically increased the accumulation of rutin in all the lines except the *FtMYB13*^*D283N*^ overexpressing line (Fig. 6A), indicating that FtMYB13^D283N^ protein is stable in response to MeJA. Immunoblot analysis of total hairy root protein with anti-HA antibodies revealed that MeJA caused a decrease in the amount of FtMYB13, FtMYB13^D281N^, and FtMYB13^D285N^, but not FtMYB13^D283N^ (Supplementary Fig. S9), indicating that the Asp 283 residue is responsible for JA-induced degradation.

Subsequently, qRT–PCR was performed to investigate the expression patterns of eight rutin biosynthesis genes (*FtPAL*, *FtC4H*, *FtCHI*, *FtF3H*, *Ft4CL*, *FtCHS*, *FtF3ʹH*, and *FtFLS*) in different *F. tataricum* hairy root lines. The transcripts of *FtPAL*, *FtC4H*, *Ft4CL*, *FtCHS*, and *FtFLS* were significantly repressed in the *FtMYB13*, *FtMYB13*^*D283N*^, *FtMYB13*^*D285N*^, *FtMYB14*, *FtMYB15*, and *FtMYB16* overexpressing lines relative to the transcript levels of WT, EV, and *FtMYB13*^*D281N*^, *FtMYB14*^*D266N*^, and *FtMYB15*^*D258N*^ overexpressing lines (Fig. 6B). Additionally, in the *FtSAD2* and *FtJAZ1* overexpressing lines, six of the eight rutin biosynthesis genes showed lower transcript levels compared with the corresponding levels in WT lines. These results demonstrate that FtMYB13, FtMYB14, FtMYB15, and FtMYB16 regulate rutin biosynthesis by repressing the gene expression of key enzymes, including *FtPAL*, *FtC4H*, *Ft4CL*, *FtCHS*, and *FtFLS,* and that this repression is modulated by FtSAD2 and FtJAZ1 via the conserved Asp residues of the SID-like motif.

## Discussion

The R2R3-MYB TFs are the main regulators of the phenylpropanoid biosynthetic pathway in plants (Du *et al.*, 2010; Zhou *et al.*, 2015*a*). It has been demonstrated that the MYB factors in the same subgroup share a similar function (Zhou *et al.*, 2015*a*). In Arabidopsis, AtMYB4, together with AtMYB7, AtMYB32, and AtMYB3, belong to the S4 MYB TFs, which act as transcriptional repressors owing to the EAR motif that they possess (Jin *et al.*, 2000; Dubos *et al.*, 2010; Fornalé *et al.*, 2014). In this study, we identified a clade of S4 MYB TFs from buckwheat, FtMYB13, FtMYB14, FtMYB15, and FtMYB16, containing the EAR repressing motif. It has been reported that the EAR motif is not essential for mediating protein–protein interaction (Zeng *et al.*, 2015). Our previous findings showed that AtMYB4, AtMYB7, and AtMYB32 possess the conserved GY/FDFLGL motif or SID motif in their C-terminus, which mediates the transport of these MYBs into the nucleus (Zhou *et al.*, 2015*a*). Deletion of the SID motif of AtMYB4 results in the loss of a loop structure in its C-terminus, which abolishes protein–protein interaction (Zhou *et al.*, 2015*a*). FtMYB13, FtMYB14, and FtMYB15 possess the conserved SID domain in their C-terminus and can interact with FtSAD2, while FtMYB16 exhibits no interaction with FtSAD2 due to the absence of a SID motif (Fig. 3). Supporting evidence for an additional function of the SID motif was provided by the fact that deletion of the SID motif of BrMYB4 leads to the loss of the capacity to repress the expression of its target gene, *BrC4H* (Zhang *et al.*, 2014).

As shown in Fig. 1C, the total *FtMYBs* mRNA exhibits the highest accumulation in root, and the lowest in leaf and flower. Interestingly, the highest content of rutin was found in leaf and flower and the lowest in root Fig. 1D, suggesting that rutin biosynthesis is spatially regulated. Further qRT–PCR analysis showed different transcript accumulation patterns of *FtMYB13*, *FtMYB14*, *FtMYB15*, and *FtMYB16* in different tissues. In roots, *FtMYB16* showed the highest expression level, while *FtSAD2* showed the lowest (Fig. 1). Therefore, the lowest accumulation of FtSAD2 protein in root may well be another explanation for the fact that FtMYB16 does not interact with FtSAD2 (Fig. 3). This strongly suggests the existence of an organ-specific signaling pathway from the lower part of the root to the upper part of the hypocotyl and to the fruit.

BLAST searching on the amino acid sequences of FtMYB13, FtMYB14, FtMYB15, and FtMYB16 reveals that the conserved SID domain has the sequence GXXDFxxxG/DL. It was previously reported that the conserved Asp (D) of the SID domain contributes to protein–protein interaction (Zhou *et al.*, 2015*a*). Therefore, the presence of two conserved Asp residues in the SID domain of the FtMYBs led us to hypothesize that the FtMYBs probably interact with one or more regulators. Indeed, the first Asp residue in the SID domain contributes to the interaction with FtSAD2. Interestingly, the second Asp residue in the SID domain of FtMYB13 contributes to its interaction with FtJAZ1, which is consistent with previous findings that the conserved Asp residue in the JAZ interacting domain of MYC2 and MYC3 plays an important role for binding to most JAZ proteins in the JA signaling pathway (Goossens *et al.*, 2015). This provides a mechanistic understanding of how the protein–protein interaction could be fine-tuned by the Asp residue of the SID motif.

The transcriptional complexes formed by protein–protein interactions between MYBs and other factors are necessary to control gene expression. For instance, the MYB–basic helix-loop-helix (bHLH)–WD40 complex is responsible for the regulation of expression of genes involved in anthocyanin biosynthesis (Zhou and Memelink, 2016). In Arabidopsis, AtJAZ proteins interact not only with bHLHs but also with MYBs, such as MYB75, which then disrupts the interactions between MYB and bHLH activators, decreasing their transcriptional activity (Qi *et al.*, 2011). On the basis of our findings, FtSAD2, as an interacting partner of FtMYB13, FtMYB14, and FtMYB15, mediates the transport of these MYBs into the nucleus, while FtJAZ1 interacts with only FtMYB13. The repression activity of FtMYB13 is significantly regulated by FtSAD2 or FtJAZ1.

The rutin biosynthesis pathway is induced by MeJA (Fig. 6A), indicating that JAs could regulate the TFs or key enzymes of this pathway at the protein or the transcript level. In this study, JAs could induce *FtMYB13*, *FtMYB14*, and *FtMYB15* expression (i.e. regulation at the transcript level). Moreover, JAs directly affected FtMYB13, FtMYB14, and FtMYB15 protein stability via the 26S proteasome pathway (Fig. 2), which established these TFs as components of JA signal transduction. These four MYB proteins share relatively high amino acid sequence identity except for the C-terminal SID motif, which is totally absent in FtMYB16. Immunoblot analysis showed that FtMYB16 and FtMYB13^D283N^ are stable after MeJA treatment, indicating that the SID motif is a unique functional domain for degradation. Our results demonstrated that the degradation of FtMYBs in response to JAs is COI1 dependent. The JA receptor COI1 binds bioactive JAs and interacts with JAZ repressors, leading to their degradation (Zhou and Memelink, 2016). The way in which these FtMYBs function as repressors could be similar to the action of JAZ on gene repression. Therefore, further research is needed to demonstrate the role of these MYB factors in JA signaling and the mechanism of signal transduction.

**Fig. 7.**
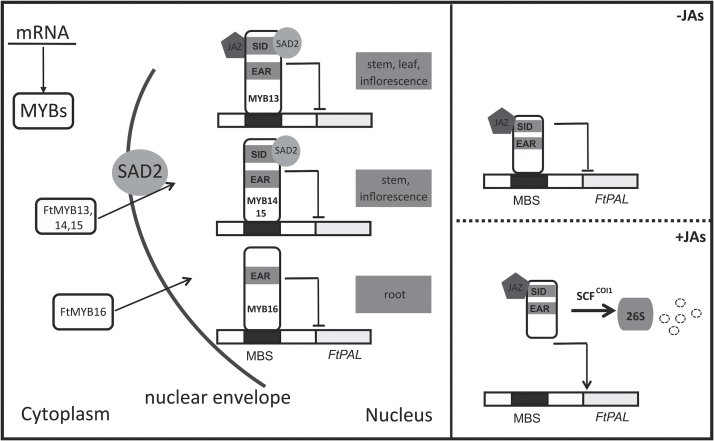
Model of spatial repression of rutin biosynthesis by JA-responsive MYB factors in *F. tataricum*. Left panel: FtMYB13, FtMYB14, FtMYB15, and FtMYB16 are synthesized in the cytoplasm. FtMYB13, FtMYB14, and FtMYB15 are recruited by an importin protein, Sensitive to ABA and Drought 2 (SAD2). FtSAD2 recognizes the nuclear pore complex, which then transports the FtSAD2/FtMYB13, FtSAD2/FtMYB14, and FtSAD2/FtMYB15 complexes into the nucleus, increasing the repression activity of FtMYB factors in stem and inflorescence. FtMYB16 specifically acts as a repressor, depending on the EAR motif, in root. In the nucleus, FtJAZ1 interacts with FtMYB13 and acts as a co-repressor to repress expression of the FtMYB13 target genes of phenylpropanoid biosynthesis via binding to MYB binding sites (MBS), mainly in stem, leaves, and inflorescence. Right panels: Simplified transcriptional networks of JA signaling in rutin biosynthesis. (Upper panel) Binding of the JAZ repressor to FtMYB TFs represses the expression of rutin biosynthesis pathway genes. (Lower panel) The presence of bioactive JAs results in COI1-mediated degradation of JAZ and FtMYB repressors via the 26S proteasome pathway, thus inducing rutin accumulation. Arrows indicate activation, and T-shaped lines indicate inhibition. SCF, Skp–Cullin–F-box protein.

In conclusion, in this study we identified a clade of JA-responsive S4 R2R3-MYB TFs—FtMYB13, FtMYB14, FtMYB15, and FtMYB16—that act as repressors of phenylpropanoid biosynthesis in buckwheat. FtMYB13, FtMYB14, and FtMYB15 can be degraded via the 26S proteasome in the COI1-dependent JA signaling pathway, and this degradation is due to the SID motif of their C-terminus. FtSAD2 directly interacts with FtMYB13, FtMYB14, and FtMYB15, and subsequently mediates the transport of these repressors into the nucleus in stem and inflorescence. Additionally, FtJAZ1 interacts with FtMYB13 and promotes the repression activity of FtMYB13, mainly in stem, leaves, and inflorescence. FtMYB16 specifically acts as a repressor, dependent on the EAR motif, in roots, while the repression activity of FtMYB13, FtMYB14, and FtMYB15 is due to the conserved Asp residues of the SID domain. Based on these data, we propose a coherent model of the spatial repression of rutin biosynthesis by JA-responsive MYB factors in *F. tataricum* (Fig. 7), which could provide valuable molecular knowledge for further plant metabolic engineering purposes.

## Supplementary data

Supplementary data are available at *JXB* online.

Fig. S1. Amino acid sequence alignment of a clade of subgroup 4 R2R3-MYB TFs from *F. tataricum* with subgroup 4 R2R3-MYB TFs from Arabidopsis.

Fig. S2. Phylogeny of R2R3-MYB TFs including two activators, FtMYB1 (JF313345) and FtMYB2 (JF313347), from *F. tataricum* with subgroup 4 R2R3-MYB TFs from Arabidopsis.

Fig. S3. Transcriptional repression activity assays of FtMYB13, FtMYB14, FtMYB15, and FtMYB16.

Fig. S4. Immunoblot analysis with anti-GFP antibodies of total protein extracts from Arabidopsis *coi-1* leaf protoplasts transiently co-expressing GFP and FtMYB13-GFP, FtMYB14-GFP, or FtMYB15-GFP.

Fig. S5. Amino acid sequence alignment of the SID domain of FtMYB13, FtMYB14, and FtMYB15 with AtMYB4, AtMYB7, and AtMYB32.

Fig. S6. Subcellular localization of FtMYB13D281N-GFP, FtMYB13D283N-GFP, FtMYB13D285N-GFP, FtMYB14D2 66N-GFP, and FtMYB15D258N-GFP in Arabidopsis protoplasts.

Fig. S7. The transcriptional activity of FtMYB14^D266N^ or FtMYB15^D258N^ was not affected by FtSAD2 or FtJAZ1.

Fig. S8. Immunoblot analysis with anti-HA antibodies of total protein extracts from *FtMYB13-HA*, *FtMYB13*^*D281N*^*-HA*, *FtMYB13*^*D283N*^*-HA*, *FtMYB13*^*D285N*^*-HA*, *FtMYB14-HA*, *FtMYB14*^*D266N*^*-HA*, *FtMYB15-HA*, *FtMYB15*^*D258N*^*-HA*, *FtMYB16-HA*, *FtSAD2-HA*, or *FtJAZ1-HA* overexpressing hairy root lines of *F. tataricum*.

Fig. S9. Immunoblot analysis with anti-HA antibodies of total protein extracts from *F. tataricum* hairy roots expressing *FtMYB13-HA*, *FtMYB13D281N-HA*, *FtMYB13D283N-HA*, and *FtMYB13D285N-HA*.

Fig. S10. Immunoblot analysis with anti-GFP antibodies of total protein extracts from Arabidopsis cell suspension protoplasts transiently co-expressing GFP and FtMYB13ΔC-GFP, FtMYB14ΔC-GFP, FtMYB15ΔC-GFP, or FtMYB16ΔC-GFP.

Table S1. List of primers used in this study.

Supplementary_Material

## Abbreviations:

CB: Common buckwheat
GFP: green fluorescent protein
JA: jasmonate
JAZ: jasmonate ZIM domain
PAL: phenylalanine ammonia lyase
qRT–PCR: quantitative real-time PCR
SAD2: Sensitive to ABA and Drought 2
SID: SAD2 Interaction Domain
TB: Tartary buckwheat
TF: transcription factor
Y1H: yeast one-hybrid
Y2H: yeast two-hybrid
YFP: yellow fluorescent protein.
