# Supplementary material for: Jasmonate-responsive MYB factors spatially repress rutin biosynthesis in Fagopyrum tataricum
Source: J Exp Bot. 2018 Jan 31;69(8):1955–66. doi: 10.1093/jxb/ery032 (PMC6018783; doi:10.1093/jxb/ery032)
Supplement: Supplementary_Material [file ery032_suppl_supplementary-figures-s1-s10_table-s1.pdf]

Supplementary Data

```
                                R2
AtMYB32   MGRSPCCEKDHTNKGAWTKEEDDKLISYIKAHGEGCWRSLPRSAGLQRCGKSCRLRWINYLRPD    64
AtMYB7    MGRSPCCEKEHMNKGAWTKEEDERLVSYIKSHGEGCWRSLPRAAGLLRCGKSCRLRWINYLRPD    64
FtMYB15   MGRSPCCEKAHTNKGAWTKEEDDRLIAYIKTHGEGCWRSLPKAAGLLRCGKSCRLRWINYLRPD    64
FtMYB14   MGRSPCCEKAHTNKGAWTKEEDDRLTAYIRAHGEGCWRSLPKAAGLLRCGKSCRLRWINYLRPD    64
FtMYB16   MGRSPCCEKEHTNKGAWSKEEDERLINYINSHGEGSWRSLPKHAGLQRCGKSCRLRWINYLRPD    64
FtMYB13   MGRAPCCSKVGLHRGPWTPKEDSLLTNYIQTHGEGCWRSLPKNAGLLRCGKSCRLRWMNYLRPD    64
FtMYB11   MASSSSRKDLDRIKGPWSPEEDDILQKLVDKHGPRNWSLISKSIP.GRSGKSCRLRWCNQLSPE    63
AtMYB3    MGRSPCCEKAHMNKGAWTKEEDQLLVDYIRKHGEGCWRSLPRAAGLQRCGKSCRLRWMNYLRPD    64
AtMYB4    MGRSPCCEKAHTNKGAWTKEEDERLVAYIKAHGEGCWRSLPKAAGLLRCGKSCRLRWINYLRPD    64
Consensus mgrspccekahtnkgawtkeeddrliayikahgegcwrslpkaagllrcgkscrlrwinylrpd
                       R3
AtMYB32   LKRGNFTLEEDDLIIKLHSLLGNKWSLIATRLPGRTDNEIKNYWNTHVKRKLLRKGIDPATHRP   128
AtMYB7    LKRGNFTHDEDELIIKLHSLLGNKWSLIAARLPGRTDNEIKNYWNTHIKRKLLSKGIDPATHRG   128
FtMYB15   LKRGNFTEEEDELIIKLHSLLGNKWSLIAGRLPGRTDNEIKNYWNTHIKRKLLSRGIDPITHRS   128
FtMYB14   LKRGNFSDEEDELIIKLHSLLGNKWSLIAGRLPGRTDNEIKNYWNTHIKRKLLSRGIDPVTHRP   128
FtMYB16   LKRGNFTQQEDDLIIDLHTLLGNKWSFIAARLPGRTDNEIKNYWNTHIKRRLLARRQSFTRKDN   128
FtMYB13   IKRGNISPDEDDLIIRLHALLGNRWSLIAGRLPGRTDNEIKNYWNTHLSKKLRSQGTDPNTHKK   128
FtMYB11   VEHRPFTPDEDAAIVQAHARFGNKWATIARMLVGRTDNAIKNHWNSTLKRKCSSMSGGDFNFDL   127
AtMYB3    LKRGNFTEEEDELIIKLHSLLGNKWSLIAGRLPGRTDNEIKNYWNTHIKRKLLSRGIDPNSHRL   128
AtMYB4    LKRGNFTEEEDELIIKLHSLLGNKWSLIAGRLPGRTDNEIKNYWNTHIRRKLINRGIDPTSHRP   128
Consensus lkrgnfteeedeliiklhsllgnkwsliagrlpgrtdneiknywnthikrkllsrgidp thrp

AtMYB32   INETKTSQDSSDSSKTEDPLVK.....ILSFG..PQLEKIANFG..DERIQKRV........EY   175
AtMYB7    INEAKIS....DLKKTKDQIVK.....DVSFV..TKFEETDKSG..DQKQNKYIRNGLVCKEER   179
FtMYB15   VNEVEVEGEVIPSPTP.TTTTSISFGATIP...KLEQD.LHDHNLMTNSRFAAIT.GSKSEERN   186
FtMYB14   LNEASSPTNTTTTTNSNTRTSSTKITTTISFASNLKQEPLLDQNIITTRLLSSSSPGSKLRSTN   192
FtMYB16   VTTTASS......ILHFAPTSN.....NIT.......TKVLHHGPVDVGVSG........HYHD   166
FtMYB13   LSDLDPIPPKPSKPKKKRTPQTNNKKQKGSDPSAQNKTKVHQPKPVRVTSLSLTRNNSNTSFES   192
FtMYB11   HANPPPLKR..SASVGPATNVSG...LNFN.PGSPSGSDISDSGGSLSHVYRPVPLSVVPSFRE   185
AtMYB3    INESVVS....PSSLQNDVVET....IHLDFSGPVKPEPVREEIGMVNNCESSG....TTSEKD   180
AtMYB4    IQESSASQ...DSKPTQLEPVTSNT.INISFTSAPKVETFHESISFPGKSEKISMLTFKEEKDE   188
Consensus ine    s     dsskt dttvs      nisf s pk ekvhdsg  d    k     gsk see

                     EAR
AtMYB32   SVVEERC....LDLNLELRISPPWQDKLHDERNLRFG..RVKYRCSACRFGFGNGKECSCNNVK   233
AtMYB7    VVVEEKIG...PDLNLELRISPPWQNQ...........REISTCTASRFYMENDMECSSETVK   228
FtMYB15   PGY......RCPDLNLELRISPPS.HQHQPEQLKSGGIIVNSNPCFKCSFGMQSGQVCKCNTFD   243
FtMYB14   SGLGFEIRDQSLDLNLDLKISPPSHHQHQPEQLKIG......VMCFKCNLGLQNSQDCSCNIFG   250
FtMYB16   SNN.........SLNLELSISLPSTSN.........................NMQEHERFTLW   195
FtMYB13   NLTSVSTP..TSQGSGGPPIVPPTYWQPYD.........LTTSGVRFLIGCEEEDDVAGLVVQ   244
FtMYB11   ITPKIE......EPETALSLSLPGFDSGEG...........SNQCSGSVDMLSQTSCPVVPEK   231
AtMYB3    YGNEED.....WVLNLELSVGPSYRYES.............TRKVSVVDSAESTRRWGSELFG   225
AtMYB4    CPVQEKF....PDLNLELRISLPDDVDRLQGHG...K..STTPRCFKCSLGMINGMECRCGRMR   243
Consensus sgvee       pdlnlelrispps   q   e            rcfkcsfgmengqecscntfk

                              SID-domain
AtMYB32   CQTEDSSSSSYSSTDISSS.IGYDFLGLNN...TRVLDF.......STLEMK.......   274
AtMYB7    CQTENSSSISYSSIDISSSNVGYDFLGLK....TRILDF.......RSLEMK.......   269
FtMYB15   SKG.NSS....ECSNIGFH....DFLGLRG...SATLDY.......RTLEMK.......   276
FtMYB14   NKT.NASN...DHSNIGIH....DFLGLRV...SANLDF.......RSLEMK.......   284
FtMYB16   RESSNAT...........................TS.......................   204
FtMYB13   EQGVMEEPLVSDQSDMLDK.LYDEYLQLIKADDDHGLDFDLDLDSDQARELDSFAESLL   302
FtMYB11   VFGPFSRELLSVMQDMIRQEVR.SYMDQQGGGTCYNQTEAISNAMMNRMGMNSVE....   285
AtMYB3    AHESDAVC...LCCRIGLF....RNESCRN...CRVSDV.......RTH..........   257
AtMYB4    CDVVGGSS...KGSDMSNG...FDFLGLAKKETTSLLGF.......RSLEMK.......   282
Consensus cqg nsss      sdig      dflglr     tr ldf        rslemk
```

Fig. S1. Amino acid sequence alignment of a clade of subgroup 4 R2R3-MYB TFs from *F. tataricum* with subgroup 4 R2R3-MYB TFs from *Arabidopsis*. Sequences were aligned using the clustalW and MEGA version 5. FtMYB11 (KU498041), FtMYB13 (KY290579), FtMYB14 (KY290580), FtMYB15 (KY290581), FtMYB16 (KY290582).

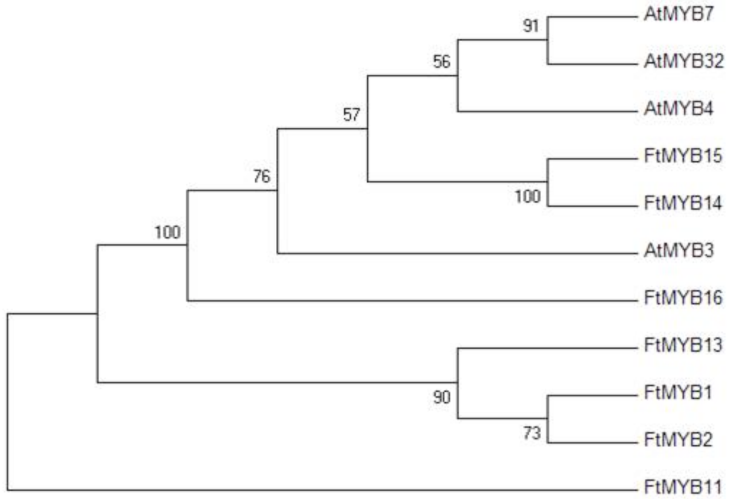

Fig. S2. Phylogeny of R2R3-MYB TFs including two activators: FtMYB1 (JF313345) and FtMYB2 (JF313347) from *F. tataricum* with subgroup 4 R2R3-MYB TFs from *Arabidopsis*. Phylogenetic tree is based on alignment of complete protein sequences.

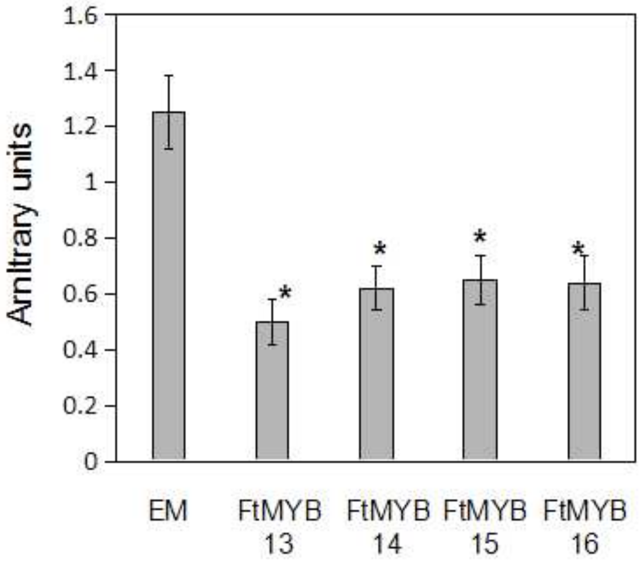

Fig. S3. Transcriptional repression activity assays of FtMYB13, FtMYB14, FtMYB15 and FtMYB16. The transformed yeasts were selected on SD/-W media and then β-galactosidase activity assays (Arbitrary units) were performed. Empty pAS2.1 vector was used as a negative control. The mean value is from three independent measurements, and error bars indicate ± SD.

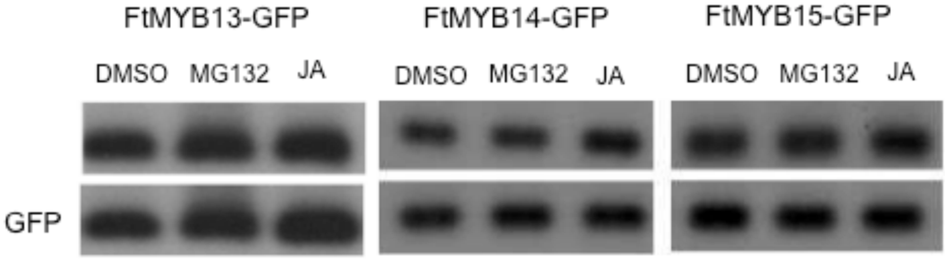

Fig. S4. Immunoblot analysis with anti-GFP antibodies of total protein extracts from Arabidopsis *coi-1* leaf protoplasts transiently co-expressing GFP and FtMYB13-GFP, FtMYB14-GFP or FtMYB15-GFP. Arabidopsis protoplasts were harvested 18 hrs after transformation of protoplasts treated for 4 hrs with the solvent DMSO at 0.1% (v/v) final concentration or with 50 μM of the 26S proteasome inhibitor MG132 or with 50 μM MeJA.

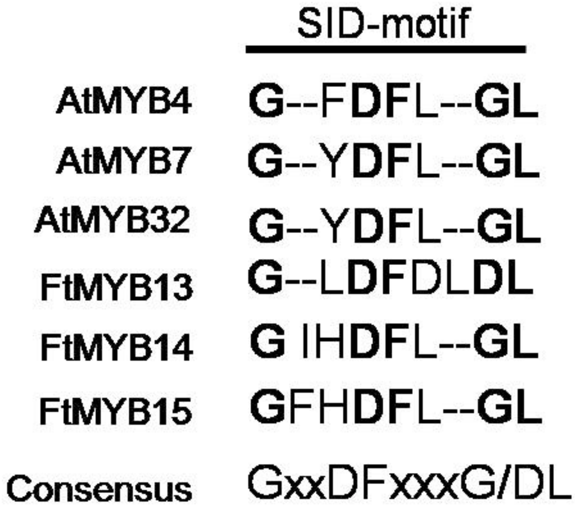

Fig. S5. Amino acid sequence alignment of the SID domain of FtMYB13, FtMYB14 and FtMYB15 with AtMYB4, AtMYB7 and AtMYB32. Sequences were aligned using the clustalW and MEGA version 5.

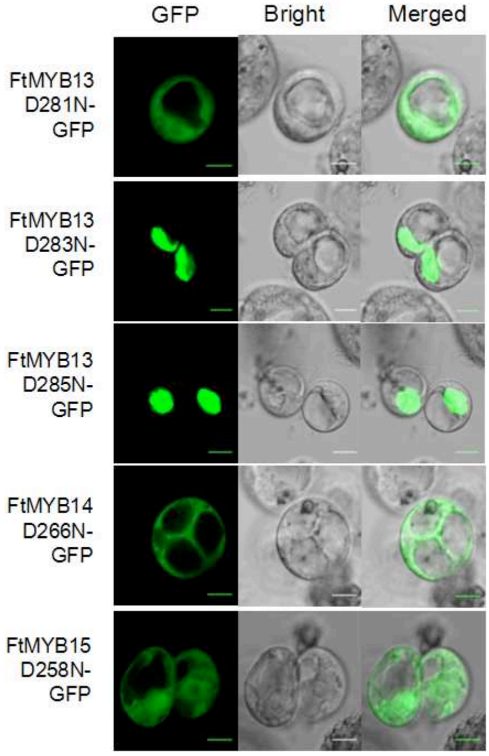

Fig. S6. Subcelluar localization of FtMYB13D281N-GFP, FtMYB13D283N-GFP, FtMYB13D285N-GFP, FtMYB14D266N-GFP or FtMYB15D258N-GFP in Arabidopsis protoplasts. Scale bar=20 μm.

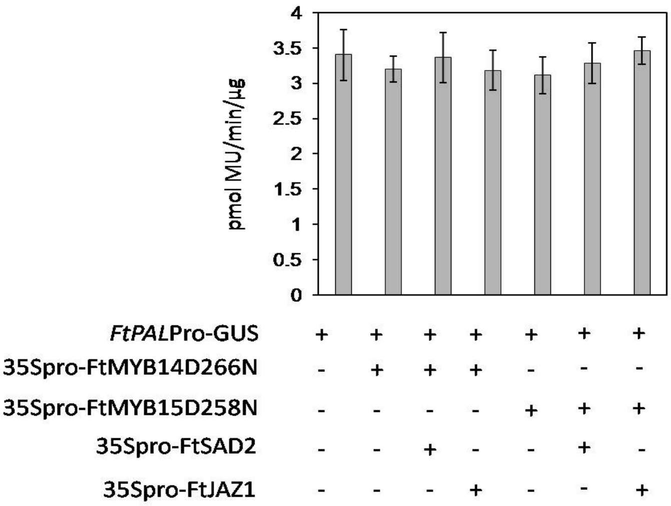

Fig. S7. The transcriptional activity of FtMYB14^{D266N} or FtMYB15^{D258N} was not affected by FtSAD2 or FtJAZ1. Arabidopsis protoplasts were co-transformed with 2μg reporter construct of *FtPALpro*-GUS and 2μg of effector plasmids. The effector constructs consisted of an expression vector carrying the CaMV 35S promoter without or with the *FtMYB14^{D266N}*, *FtMYB15^{D258N}*, *FtSAD2* or *FtJAZ1* genes. Values represent means ± SE of triplicate experiments.

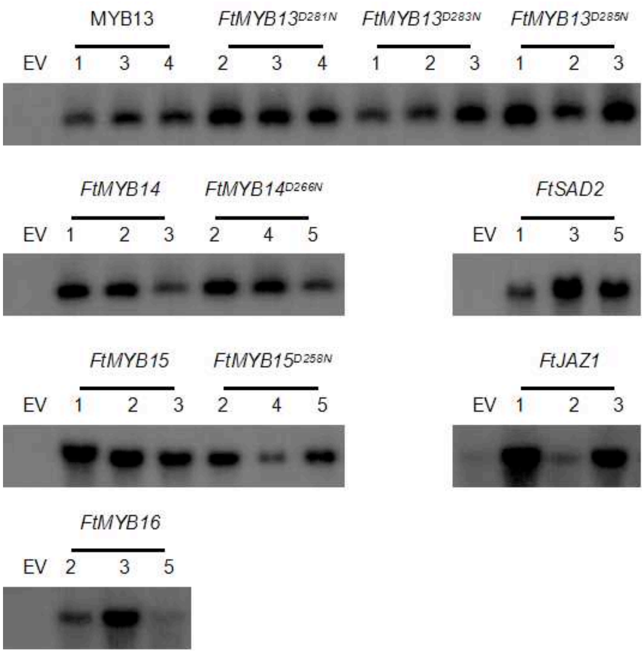

Fig. S8. Immunoblot analysis with anti-HA antibodies of total protein extracts from *FtMYB13-HA*, *FtMYB13^{D281N}-HA*, *FtMYB13^{D283N}-HA*, *FtMYB13^{D285N}-HA*, *FtMYB14-HA*, *FtMYB14^{D266N}-HA*, *FtMYB15-HA*, *FtMYB15^{D258N}-HA*, *FtMYB16-HA*, *FtSAD2-HA* or *FtJAZ1-HA* overexpressing hairy root lines of *F. tataricum* as indicated. 5 µg protein were loaded for each sample.

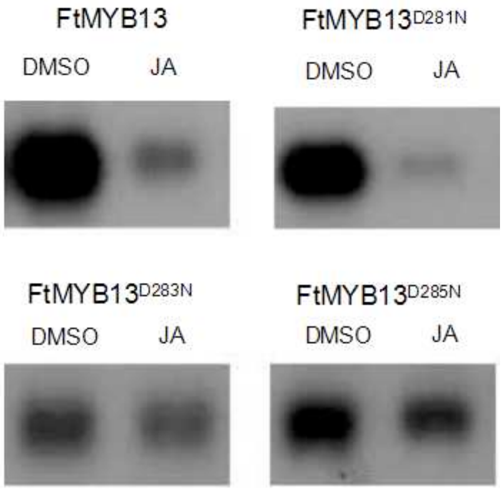

Fig. S9. Immunoblot analysis with anti-HA antibodies of total protein extracts from *F. tataricum* hairy roots expressing *FtMYB13-HA*, *FtMYB13D281N-HA*, *FtMYB13D283N-HA* and *FtMYB13D285N-HA*. The 20 days hairy root lines were harvested 12 hrs after treated with the solvent DMSO at 0.1% (v/v) final concentration or with 50 µM MeJA. 5 µg protein were loaded for each sample.

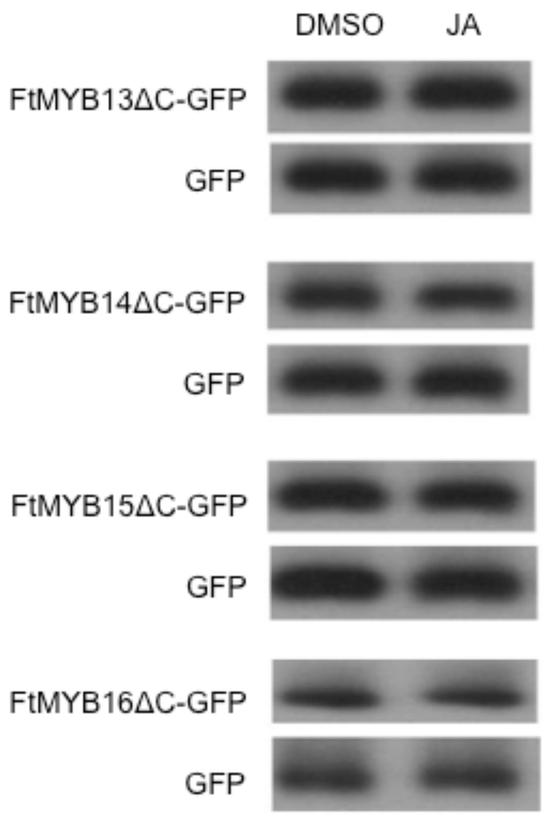

Fig. S10 Immunoblot analysis with anti-GFP antibodies of total protein extracts from Arabidopsis cell suspension protoplasts transiently co-expressing GFP and FtMYB13ΔC-GFP, FtMYB14ΔC-GFP, FtMYB15ΔC-GFP or FtMYB16ΔC-GFP. Arabidopsis protoplasts were harvested 18 hrs after transformation of protoplasts treated for 4 hrs with the solvent DMSO at 0.1% (v/v) final concentration or with 50 μM MeJA.

Table S1. List of primers used in this study.

Genes	Primers
Primers used for qRT-PCR	
FtMYB13	Fw: 5'-GCCGTACGATCTGACGACGAG-3'<br>Rv: 5'-GTCCATGATCATCATCCGC-3'
FtMYB14	Fw: 5'-CGAGATAAGAGATCAAAGC-3'<br>Rv: 5'-CTATGATCATTACTAGCAT-3'
FtMYB15	Fw: 5'-CATCAGCCAGAACAACTG-3'<br>Rv: 5'-CATGGAAGCCAATGTTGC-3'
FtMYB16	Fw: 5'-ACGTCGGAGTATCCGGTC-3'<br>Rv: 5'-GCTTGTTGTGGCATTAG-3'
FtSAD2	Fw: 5'-GATCCTGTGGAGCAATCTAAG-3'<br>Rv: 5'-AGACCCTGCCATTGCTCG-3'
FtH3	Fw: 5'-GAAATTCGCAAGTACCAGAAGAG-3'<br>Rv: 5'-CCAACAAGGTATGCCTCAGC-3'
Primers used for yeast two hybrid	
FtMYB13	ADFw: 5'-GCCCCGGGGATGGGAAGAGCTCCTTGTTGC-3'<br>ADRv: 5'-AGCTCGAGTCAGATGAGCAAAGACTCAGC-3'
FtMYB14	ADFw: 5'-GCCCCGGGGATGGGTAGATCTCCATGTTGTG-3'<br>ADRv: 5'-AGCTCGAGTCATTTCATCTCCAATGATC-3'
FtMYB15	ADFw: 5'-GCCCCGGGGATGGGTCGATCTCCATGTTGC-3'<br>ADRv: 5'-AGCTCGAGTCATTTCATCTCCAAAGTTCTATAG-3'
FtMYB16	ADFw: 5'-GCCCCGGGGATGGGGAGATCACCTTGCTGC-3'<br>ADRv:5'-AGCTCGAGCTAGCTTGTTGTGGCATTAGAAG-3'
FtSAD2	BDFw: 5'-CTCATATGGATCTTCCAAGCCTCGCT-3'

	BDRv: 5’-CCGTCGACTCAAGAGAGTTCTTCGAGCAT-3’
FtJAZ1	ABDFw: 5’-TGCCATGGAGATGAACTTGTTCCCACTGAAAG-3’<br><br>ABDRv: 5’-CGGGATCCTCAGGGCTGAATCGACGTCCG-3’
FtJAZ2	ABDFw: 5’-CTCATATGATGGTGAATTTGTCGGCTTCC-3’<br><br>ABDRv: 5’-CCGTCGACTCAAGAACGTGGATTAGCAGC-3’
Primers used for yeast one hybrid	
FtMYB13	BDFw: 5’-CTCATATGGGAAGAGCTCCTTGTTGC-3’<br><br>BDRv: 5’-CCGTCGACTCAGATGAGCAAAGACTCAGC-3’
FtMYB14	BDFw: 5’-CTCATATGGGTAGATCTCCATGTTGTG-3’<br><br>BDRv: 5’-CCGTCGACTCATTTCATCTCCAATGATC-3’
FtMYB15	BDFw: 5’-CTCATATGGGTCGATCTCCATGTTGC-3’<br><br>BDRv: 5’-CCGTCGACTCATTTCATCTCCAAAGTTCTATAG-3’
FtMYB16	BDFw: 5’-CTCATATGGGGAGATCACCTTGCTGC-3’<br><br>BDRv: 5’-CGGGATCCCTAGCTTGTTGTGGCATTAGAAG-3’
FtPALpro	Fw: 5’-CGGCGGCCGCGTCGTTAAATATCGTTAAAAT-3’<br><br>Rv: 5’-TGCCCGGGCCACCCCAACGGATCCTGCAC-3’
Primers used for protoplast transactivation assays	
FtMYB13-HA	RT101Fw: 5’-AGCTCGAGATGGGAAGAGCTCCTTGTTGC-3’<br><br>RT101Rv: 5’-TGTCTAGATTAGGCGTAGTCAGGCACGTCGTAAGG GATGAGCAAAGACTCAGC-3’
FtMYB14-HA	RT101Fw: 5’-AGCTCGAGATGGGTAGATCTCCATGTTGTG-3’<br><br>RT101Rv: 5’-TGTCTAGATTAGGCGTAGTCAGGCACGTCGTAAGG TTTCATCTCCAATGATC-3’
FtMYB15-HA	RT101Fw: 5’-AGCTCGAGATGGGTCGATCTCCATGTTGC-3’<br><br>RT101Rv: 5’-TGTCTAGATTAGGCGTAGTCAGGCACGTCGTAAGG

	TTTCATCTCCAAAGTTCTATAG-3’
FtMYB16-HA	RT101Fw: 5’-AGCTCGAGATGGGGAGATCACCTTGCTGC-3’<br>RT101Rv: 5’-TGTCTAGATTAGGCGTAGTCAGGCACGTCGTAAGG GCTTGTTGTGGCATTAGAAG-3’
FtSAD2-HA	RT101Fw: 5’-AGCTCGAGATGGATCTTCCAAGCCTCGCT-3’<br>RT101Rv:5’-TGTCTAGATTAGGCGTAGTCAGGCACGTCGTAAGGGT CAAGAGAGTTCTTCGAGCAT-3’
FtJAZ1-HA	RT101Fw: 5’-AGCTCGAGATGAACTTGTTCCCACTGAAAGAG-3’<br>RT101Rv:5’-CCGGTACCTCAGGGCTGAATCGACGTCCG-3’
FtPALpro	GUSXXFw: 5’-CGGGATCCGTCGTTAAATATCGTTAAAAT-3’<br>GUSXXRv: 5’-TGCCATGGCCACCCCAACGGATCCTGCAC-3’
Primers used for BiFC	
FtMYB13	pRTL2-HAYCFw: 5’-CCGTCGACAATGGGAAGAGCTCCTTGTTGC-3’<br>pRTL2-HAYCRv: 5’-GCAAGCGGCCGCGATGAGCAAAGACTCAGC-3’<br>GFPFw: 5’- TCGTCGACATGGGAAGAGCTCCTTGTTGC-3’<br>GFPΔNFw: 5’- TCGTCGACATGTCGGATCAGAGTGACATG-3’<br>GFPΔCRv: 5’- TCGTCGACCATGTCACTCTGATCCGACAT-3’<br>GFPRv: 5’- TCGTCGACGATGAGCAAAGACTCAGC-3’
FtMYB14	pRTL2-HAYCFw: 5’-CCGTCGACAATGGGTAGATCTCCATGTTGTG-3’<br>pRTL2-HAYCRv: 5’-GCAAGCGGCCGCTTTCATCTCCAATGATC-3’<br>GFPFw: 5’- TCGTCGACATGGGTAGATCTCCATGTTGTG-3’<br>GFPΔNFw: 5’- TCGTCGACATGTTAGGGTTGCAAAACAGC-3’<br>GFPΔCRv: 5’-TCGTCGACGCTGTTTTGCAACCCTAACAT-3’<br>GFPRv: 5’- TCGTCGACTTTCATCTCCAATGATC-3’

FtMYB15	pRTL2-HAYCFw: 5’-CCGTCGACA ATGGGTCGATCTCCATGTTGC-3’<br>pRTL2-HAYCRv: 5’-GCAAGCGGCCGC<br>TTTCATCTCCAAAGTTCTATAG-3’<br>GFPFw: 5’- TCGTCGACATGGGTCGATCTCCATGTTGC-3’<br>GFPΔNFw: 5’- TCGTCGACATGAGTTTTGGTATGCAAAGTG-3’<br>GFPΔCRv: 5’-TCGTCGACCACTTTGCATACCAAAACTCAT-3’<br>GFPRv: 5’- TCGTCGAC TTTCATCTCCAAAGTTCTATAG-3’
FtMYB16	pRTL2-YNEEFw: 5’- CCGGACTAGT<br>ATGGGGAGATCACCTTGCTGC-3’<br>pRTL2-YNEERv: 5’- CGGGATCC<br>CTAGCTTGTTGTGGCATTAGAAG-3’<br>GFPΔNFw: 5’- TCGTCGACATGGTTGACGTCGGAGTATCC-3’<br>GFPΔCRv: 5’-TCGTCGACGGATACTCCGACGTCAACCAT-3’<br>GFPRv: 5’- TCGTCGACGCTTGTTGTGGCATTAGAAG-3’
FtSAD2	pRTL2-HAYCFw: 5’-CCGTCGACAATGGATCTTCCAAGCCTCGCT-3’<br>pRTL2-HAYCRv:<br>5’-GCAAGCGGCCGCGTCAAGAGAGTTCTTCGAGCAT-3’
FtJAZ1	pRTL2-HAYCFw:<br>5’-CCGTCGACAATGAACTTGTTCCCACTGAAAGAG-3’<br>pRTL2-HAYCRv:<br>5’-GCAAGCGGCCGCGTCAGGGCTGAATCGACGTCCG-3’
